# Supplementary material for: Concept and demonstration of a low-cost compact electron microscope enabled by a photothermionic carbon nanotube cathode
Source: Nat Commun. 2025 Aug 28;16:8067. doi: 10.1038/s41467-025-63413-2 (PMC12394587; doi:10.1038/s41467-025-63413-2)
Supplement: Supplementary file 1 — Supplementary Information File [file 41467_2025_63413_MOESM1_ESM.pdf]

SUPPLEMENTARY INFORMATION for

Concept and Demonstration of a Low-cost Compact Electron Microscope Enabled by a Photothermionic Carbon Nanotube Cathode

Casimir Kuzyk[1,2], Alexander Dimitrakopoulos[1,2], Alireza Nojeh[1,2,*]

[1]Department of Electrical and Computer Engineering, The University of British Columbia, Vancouver BC, V6T 1Z4, Canada

[2]Quantum Matter Institute, The University of British Columbia, Vancouver BC, V6T 1Z4, Canada

[*]Corresponding author: alireza.nojeh@ubc.ca

Carbon nanotube forest growth

The carbon nanotube (CNT) forests were grown in-house by chemical vapour deposition (CVD). A low-resistivity (0.001-0.005 Ω·cm) Si wafer was used as the growth substrate. 10 nm of Al_2O_3 and 1 nm of Fe were deposited on the substrate. The coated wafer was diced into ~5x5 mm^2 squares before being placed in the CVD reaction vessel. The vessel was first purged with argon before ramping up the temperature to 750 ^oC for the annealing stage, where a flow of argon and hydrogen gas enables the formation of small iron islands that act as catalysts for nanotube growth. Next, the high temperature was maintained while a flow of ethylene gas was introduced, which decomposes to form the multi-walled CNTs. This is a well-established process for growing CNT forests of millimeter heights–more detail can be found in reference [S1].

Vacuum system

Specimen flexibility

We present two sets of images that highlight the user-friendliness of the system and its tolerance to various specimen conditions. In figure S1 we compare images of cardboard with a sputtered metal coating, with no coating, and after being submerged in water directly before imaging. We note that images of fair quality were obtained with the uncoated, non-conductive cardboard specimen, despite some charging artifacts. Of particular interest is the wet specimen, where the sample preparation was simple, yet it resulted in a significant image quality improvement compared to the uncoated specimen. Once the small wet cardboard specimen is introduced into

the sample chamber, presumably most of the water rapidly evaporates, but it appears that a conductive residue remains on the specimen, resulting in improved image quality.

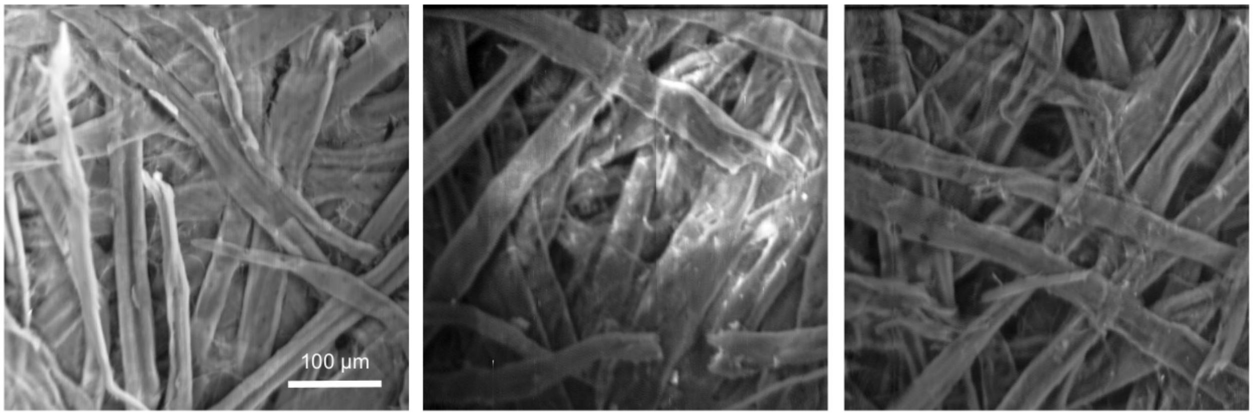

Figure S1. Effect of coating on imaging of an insulating specimen. Comparison of (left) metal coated, (middle) uncoated, and (right) wet cardboard.

The images shown in figure S2 are of a fresh maple leaf that was harvested outside of our laboratory mere minutes before imaging. Other than cutting the specimen to an appropriate size for the sample chamber, no processing was necessary. Similar to the wet cardboard specimen, charging artifacts were negligible in these images. The ease with which specimens can be imaged in this microscope is comparable to an optical microscope.

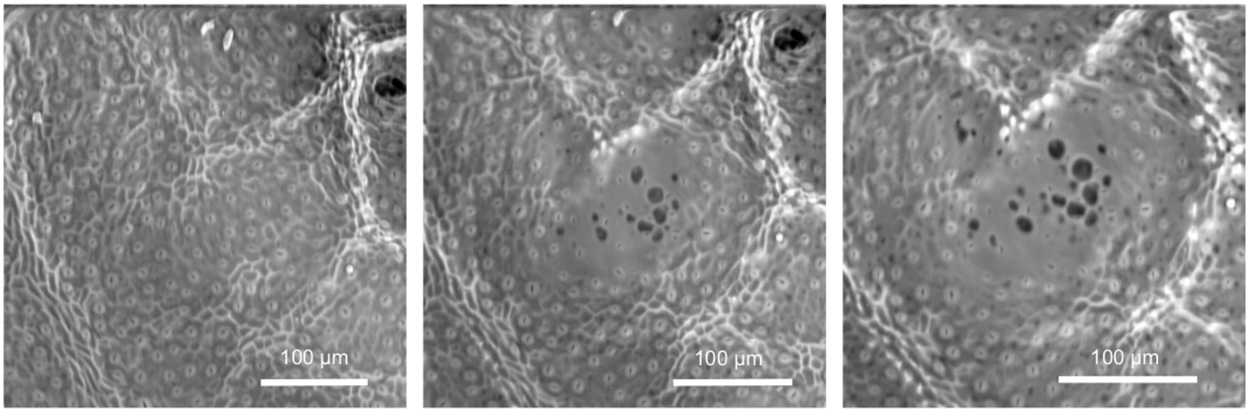

Figure S2. Imaging of a biological specimen without any sample preparation. Fresh maple leaf harvested only minutes before imaging. The progression from left to right shows damage to the sample over the course of 10 minutes from exposure to the electron beam, particularly in the middle of the sample where the exposure was increased when zooming in.

Pumpless/sealed-off operation

As discussed in the main text, an ultimate goal would be to develop a pumpless system. As a proof of concept, we inserted a soft copper connection in the vacuum line, pumped the instrument down, baked it at <100 ºC for several hours, and pinched-off the copper connection. For this particular test, a tin-sphere-on-carbon calibration standard had been placed within the end of the column, and we were able to image it at an electron beam energy of 30 keV. This sealed-off system maintained vacuum for only about an hour, but this was due to the rough nature of the various seals on this prototype and the minimal cleaning and bake-out employed in this experiment. For constructing a permanent device, established manufacturing technologies of cathode ray tube television sets are adequate. Modern examples of sealed-off devices involving carbon nanotube electron emitters are reported in references [S2-S4].

For a practical pumpless instrument, as stated in the main text, an electron-transparent window must be included at the end of the column to allow the electrons to exit and strike the specimen in its natural environment. We have experimented with silicon nitride membranes of various thicknesses as the window. A wider window facilitates electronic scanning of a wider area on the specimen, and we have observed that 50-nm-thick membranes as wide as 500 µm can withstand the requisite 1-atmosphere pressure differential. The effect of electron scattering in rough vacuum on imaging is shown in figure S3. The effect of electron scattering in the silicon nitride membrane is also shown in figure S3, where there is a decrease in contrast due to the increased background noise from the scattered electrons, as discussed in the main text.

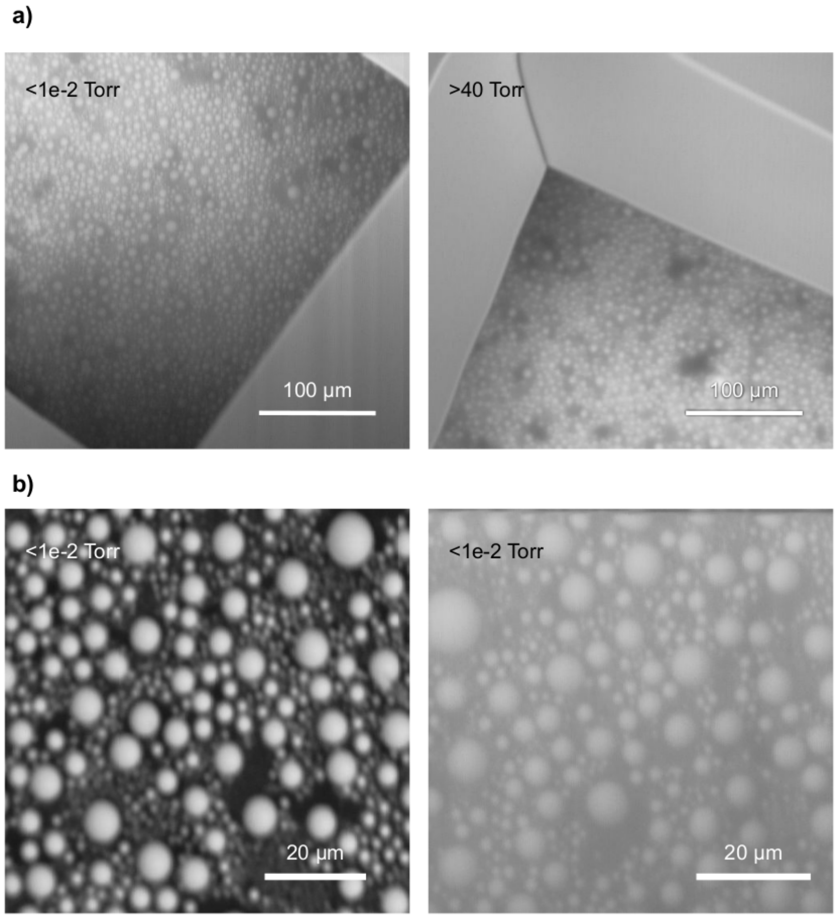

Figure S3. Effect of vacuum level and electron-transparent membrane. a) Tin-on-carbon calibration standard imaged in (left) $<10^{-2}$ Torr vacuum and (right) >40 Torr. In both cases, a 50-nm-thick silicon nitride membrane separated the electron column from the specimen. The large rectangular structure seen in the images is the silicon frame of the nitride membrane. b) Tin-on-carbon calibration standard imaged (left) directly and (right) through a 50-nm-thick silicon nitride membrane. The instrument's contrast and brightness settings were kept the same for both images.

Electron-optical system

Objective lens

The ring magnets we used for the objective lens were stock components, and thus not manufactured with high tolerances; they even had visible defects, leading to aberrations such as astigmatism. Smaller-diameter ring magnets are expected to have less circular distortion; they also provide a more concentrated magnetic field and thus a smaller focal length and better focusing. However, smaller-diameter magnets impose size limitations on the sample, especially if the focal plane is within the bore of the lens. It should also be noted that demagnetization was

observed when the lens was subject to electron beam radiation, making it impractical to place smaller-diameter lenses within the column itself (unless they could be adequately covered). An imaging performance comparison among ring magnets with different sizes is shown in figure S4. It can be seen that the instrument can operate well with a wide range of magnets–the exact choice depends on the builder’s preference and use case.

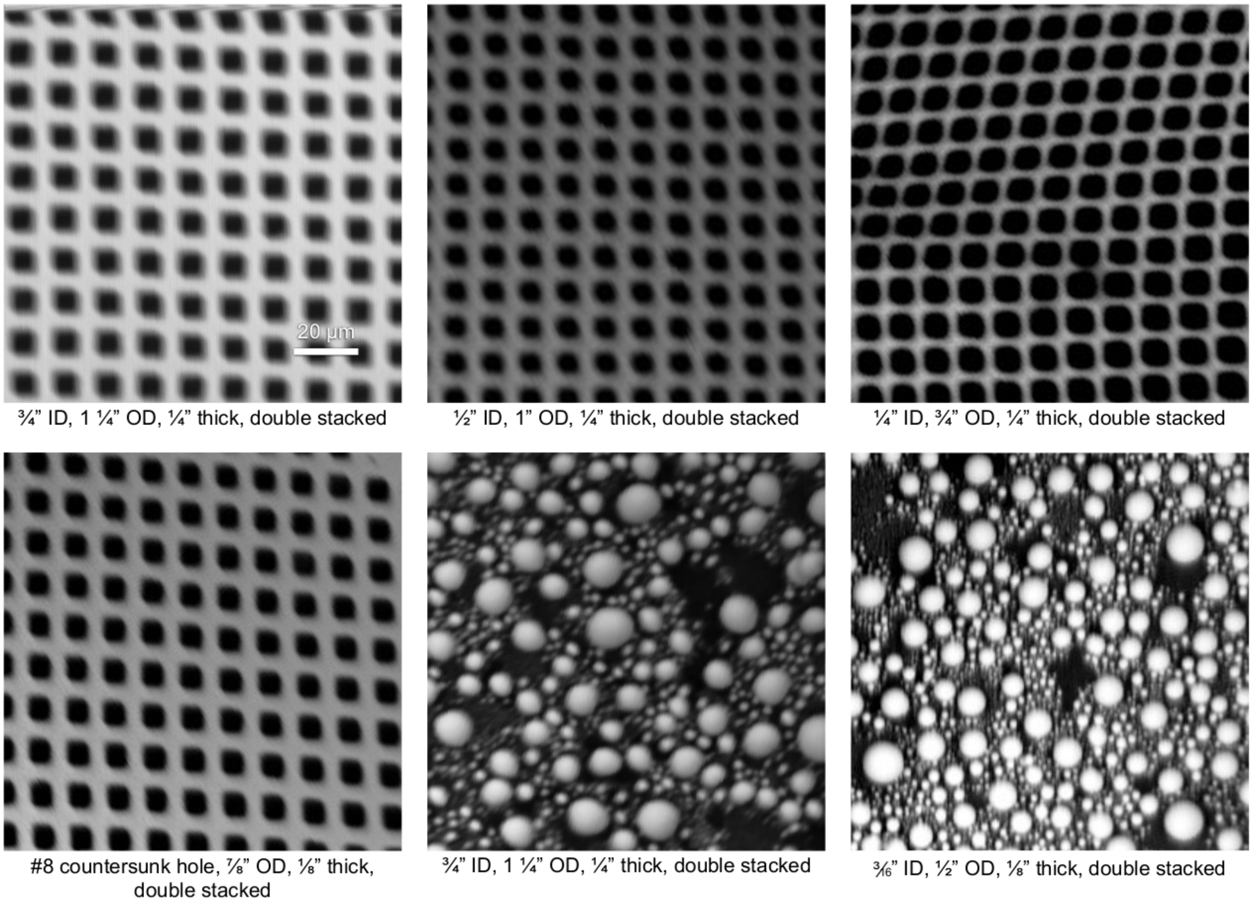

Figure S4. Flexibility of choice of permanent magnet for the objective lens. Imaging comparison using different sizes of neodymium ring magnets. The first 4 images are of a metallic 2000 mesh, and the remaining 2 images are of a tin-on-carbon calibration standard. (ID: inner diameter; OD: outer diameter)

Electron-optics improvements

A number of future improvements to the electron-optical system can be envisioned. Better focusing of the excitation laser and thus a more confined Heat Trap spot on the CNT forest surface would lead to a smaller emission area, and thus a smaller probe size and higher resolution. By using a beam expander and a focusing lens with a smaller focal length, one could

decrease the size of the Heat Trap commensurately. This, however, would require better mechanical precision to position the laser focal spot at the surface of the CNT forest, given the decreased depth of focus. The resolution could also be improved by adding a condenser lens, either by creating a magnetic circuit from the secondary field of the existing objective lens, or in the form of an additional ring magnet placed further up the beam column. This would decrease the total probe current and may require further improvements to the electron detector and amplifier circuit to utilize the weaker signal. We expect that these improvements would allow the electron probe size to be reduced to <50 nm.

While we did not use a stigmator for the specimen images presented in the main text, we have observed a significant improvement in image quality when using a stigmator, as shown in figure S5.

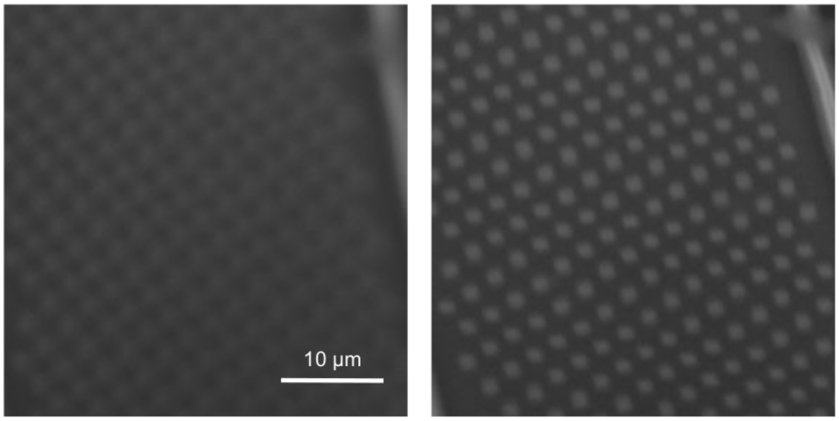

Figure S5. Effect of stigmation. Imaging comparison of an array of rectangles on a silicon wafer with (left) the stigmator off and (right) the stigmator on. Sample provided by Lumiense Photonics Inc.

Electromagnetic noise and shielding

A primary source of noise present in the images generated by the current prototypes is 60 Hz environmental noise. In order to study the impact of low-frequency magnetic field noise on the electron beam, an aluminum shield lined with a thin layer of mu metal was used. Examples of imaging with and without the shield are shown in figure S6. The power circuitry for the electromagnets was designed with high power supply rejection ratios, to minimize the effect of noise from the device itself. It should be noted that the images shown in the main text were taken without the mu-metal shielding.

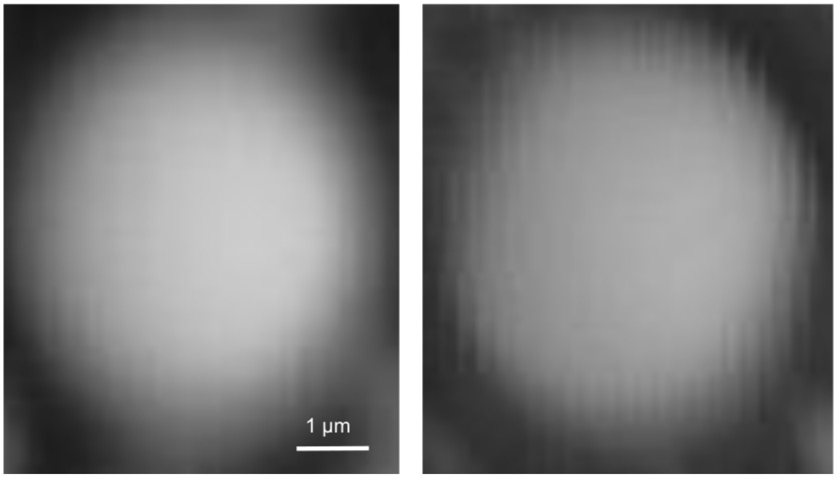

Figure S6. Effect of electromagnetic noise. Imaging comparison of a ~5-μm-diameter tin sphere (left) with and (right) without the mu-metal shielding. 60 Hz environmental noise appears as periodic, nearly vertical stripes.

Safety

The carbon nanotube forest is inside the vacuum environment of the electron source compartment. A shield covers the laser and electron source assembly to prevent stray laser reflections. The high-voltage lead of the power supply is covered by a thick layer of high-voltage insulating putty, which is surrounded by a grounded metallic cylinder, before entering the vacuum chamber using a ceramic-insulated feedthrough rated to 50 kV. X-ray generation by the high-energy electrons is expected to be minimal since the emission current is of the order of only a few microamperes, but precautions are nonetheless necessary. The main areas of X-ray generation are expected to be at the anode plate, objective aperture, and specimen, which are collectively struck by the majority of the electrons. X-rays would be significantly attenuated by the stainless steel beam column and the housing, but shields around the source assembly and beam column ensure that all possible X-ray escape paths are blocked. (The laser/X-ray covers are not shown on the instrument photo in figure 2 of the main text.) During experiments we used a radiologist's X-ray sensor for monitoring, and recorded doses were always significantly below allowed safe limits. The situation may vary in different implementations of the instrument. All appropriate safety measures relevant to nanomaterial handling, lasers, high voltage, and X-ray radiation, in addition to general electrical and mechanical safety, must be taken in implementing and using any embodiment of the concept.

Additional micrographs

We have imaged a variety of specimens with our prototypes, from biological curiosities to engineered materials. Figure S7 includes more examples of images taken with this instrument.

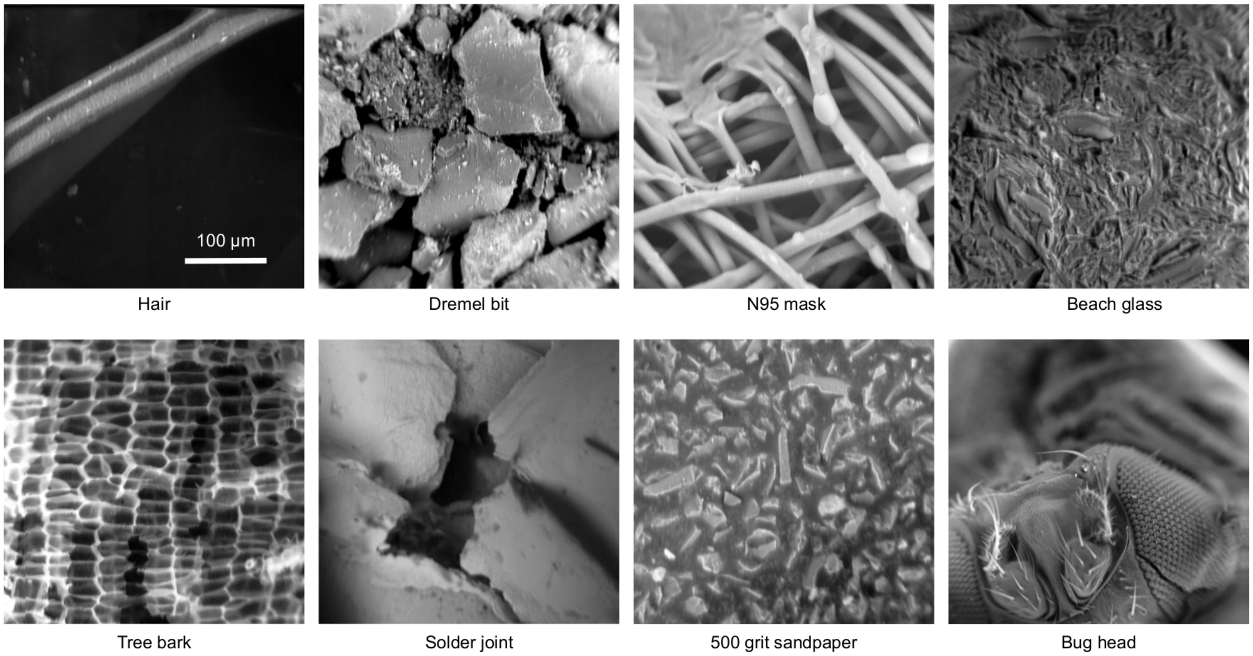

Figure S7. Various specimen images. A selection of additional micrographs obtained using this SEM, which highlight its broad range of applicability (scale bar approximate).

References

S1. Voon, K. *Modelling contributing factors to heat trapping in carbon nanotube forests* PhD thesis (University of British Columbia, Vancouver BC, Canada, 2021).

S2. Yaghoobi, P., Vahdani Moghaddam, M. & Nojeh, A. Solar electron source and thermionic solar cell. *AIP Advances* **2**, 042139 (2012).

S3. Yuan, X. *et al.* A fully-sealed carbon-nanotube cold-cathode terahertz gyrotron. *Scientific Reports* **6**, 32936 (2016).

S4. Han, J. S. *et al.* High-performance cold cathode X-ray tubes using a carbon nanotube field electron emitter. *ACS Nano* **16**, 10231–10241 (2022).
